# Biological Activity of Selected Natural and Synthetic Terpenoid Lactones

**DOI:** 10.3390/ijms22095036

**Published:** 2021-05-10

**Authors:** Alicja K. Surowiak, Lucyna Balcerzak, Stanisław Lochyński, Daniel J. Strub

**Affiliations:** 1 Department of Chemical Biology and Bioimaging, Wroclaw University of Science and Technology, Wyb. Wyspiańskiego 27, 50-370 Wroclaw, Poland; alicja.surowiak@pwr.edu.pl (A.K.S.); lucyna.balcerzak@pwr.edu.pl (L.B.); stanislaw.lochynski@pwr.edu.pl (S.L.); 2 Institute of Cosmetology, Wroclaw College of Physiotherapy, T. Kościuszki 4, 50-029 Wroclaw, Poland

**Keywords:** terpenoid, lactones, biological activity

## Abstract

Terpenoids with lactone moieties have been indicated to possess high bioactivity. Certain terpenoid lactones exist in nature, in plants and animals, but they can also be obtained by chemical synthesis. Terpenoids possessing lactone moieties are known for their cytotoxic, anti-inflammatory, antimicrobial, anticancer, and antimalarial activities. Moreover, one terpenoid lactone, artemisinin, is used as a drug against malaria. Because of these abilities, there is constant interest in new terpenoid lactones that are both isolated and synthesized, and their biological activities have been verified. In some cases, the activity of the terpenoid lactone is specifically connected to the lactone moiety. Recent works have revealed that new terpenoid lactones can demonstrate such functions and are thus considered to be potential active agents against many diseases.

## 1. Introduction

The dynamic development of the world has caused new threats to human health and wellbeing. Due to climate change and overpopulation, as a society, we face many new problems from cancer-related or pathogenic diseases that are untreatable in the traditional way. Additionally, increased demand for food has led to the progressive development of agriculture. With the development of cultivation techniques, there have also been changes in the insects that threaten them. Moreover, the environment is becoming increasingly polluted, and alternatives for protection measures are being sought. Among pesticides, antifeedants play an important role in protecting crops from herbivores. The aim of feeding deterrents is not to eliminate the pest but to reduce the quantity of the crops being destroyed below the economic harmfulness threshold. Recently, research has once again begun to focus on substances that occur in nature, as scientists are becoming inspired by nature. Lactones have been known since the 19th century and were first obtained synthetically [1]. The characteristic structure of lactones is an oxygen atom double-bonded to a carbon atom, which is attached to another oxygen atom that is part of a closed ring; thus, lactones are derivatives of various organic hydroxy acids, depending on the position of the hydroxyl group, four (β), five (γ) or six-membered (δ) rings can be possessed [2,3]. Many ester synthesis methods can be applied to synthesize lactones. First, lactones can be synthesized by the Baeyer–Villiger reaction, based on the exposure of ketones to peracids. The result is the inclusion of oxygen among alkyl or aryl substituents and carbonyl moieties, forming the corresponding ester [4]. Another method is halolactonization, where the ring is formed by the addition of oxygen and a halogen across a C=C double bond. The halogen can then be removed by free radical hydrogenation [4,5]. The subsequent reaction that is applied in lactone synthesis is the Favorskii rearrangement [4]. Additionally, there are a variety of microorganisms that can perform de novo biosynthesis of lactones, but enzymes are also known for the lactonization process [2]. Baeyer–Villiger oxidation has been applied in enzymatic protocols [4]. Two classes of enzymes are responsible for this reaction: Baeyer–Villiger monooxygenases (BVMOs) and hydrolases that follow the perhydrolase pathway. Another approach is the oxidative lactonization of diols, mostly using alcohol dehydrogenases as catalysts [6]. In nature, cyclic esters are plant secondary metabolites that are connected with catabolic processes involving structurally related fatty acids and often possess low molecular weights [2]. They are responsible for the aroma of plants, among other features, and are often constituents of essential oils [3,4]. Natural lactones are mostly γ-lactones (in plants) and δ-lactones (in animals) [2]. Lactones have also been reported in all plant parts, including berries, stone fruits, and plant food products such as wines and spirits [7]. The biosynthesis of lactones is complex and still not fully understood [2]. Many lactones present a wide range of desired biological properties [8,9] and the molecular mechanisms of their actions were, in many cases, investigated. The most important mechanism induced by terpenoid lactones is inhibition of the expression of NF-κB (nuclear factor kappa-light-chain-enhancer of activated B cells) which is directly related to anti-inflammatory and anticancer activities [10,11]. Parthenolide inhibits NF-κB by alkylation of Cys-38 in p65, Helenalin A by p65 alkylation, costunolide, triptolide and nepalolide A by IκB phosphorylation, artemisinin, triptolide, and ginkgolides by DNA binding, artemisolide by IKKβ inhibition on Cys-179, ergolide and zerumbone by IκB degradation, and elephantopins by IKK inhibition [11]. Many drugs contain this moiety and are used to treat inflammation, cancer, malaria, etc. Other lactones are in clinical trials [8]. Therefore, we decided to review recent information about lactones belonging to the terpenoid group and their biological activities. We focused our interest on lactones of both synthetic and natural origins.

## 2. Antimicrobial Activity

**Figure 1:**
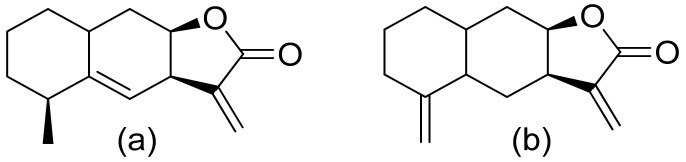
Lactones from sunflowers (**a**,**b**).

Various terpenoid lactones of both natural and synthetic origin possess antimicrobial activities. Pickman et al. reported that the sesquiterpenoid lactones from sunflowers, alantolactone and isoalantolactone (Figure 1), are active against the fungi *Sclerotinium sclerotiorum* and *Verticillium dahliae*, which are responsible for verticillium wilt, and the results were satisfactory [12]. In another study, it was indicated that both sunflower lactones have great antifungal activity against isolates of *Leptosphaeria maculans*, *Verticillium albo-atrum*, and *Fusarium graminearum* with low MIC (minimal inhibitory concentration) values ranging from 1 to 5 ppm [13]. This might be related to lipophilic character of its structures that facilitate their penetration through the cell wall [14]. 

**Figure 2:**
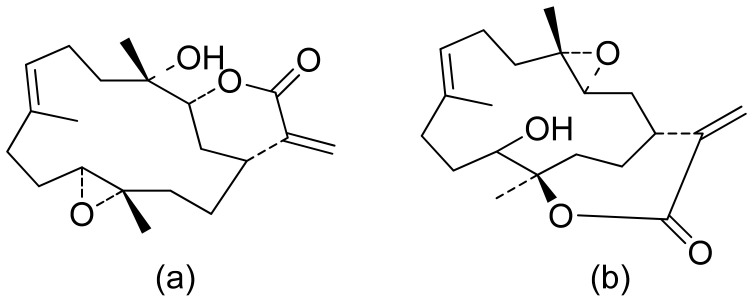
Lactones from soft coral possessing an α-methylene-lactone moiety (**a**,**b**).

The Villarreal group reported that lactones from *Asteraceae* species have both antibacterial and anti-yeast actions. They tested various lactones from the germacrolide, heliangolide, and eremophilanolide groups against *Escherichia coli* (ATCC8937), *Pseudomonas aeruginosa* (ATCC9027), *Staphylococcus aureus* (ATCC6538), and *Candida albicans* (ATCC10231). The results proved that almost all tested lactones were good growth inhibitors with MIC values ranging from 50–400 µg/mL [15]. There are also active lactones among terpenoids from the soft coral genus *Sinularia* [16]. Flexibilide and sinulariolide (Figure 2) both possess an α-methylene-lactone moiety. Both showed activity against *Bacillus subtilis* and *S. aureus*, although they were not active against fungi and Gram-negative bacteria. The authors indicated that the activity of those compounds is related to the alkylating centers or hemiacetal moieties in the molecule [17].

**Figure 3:**
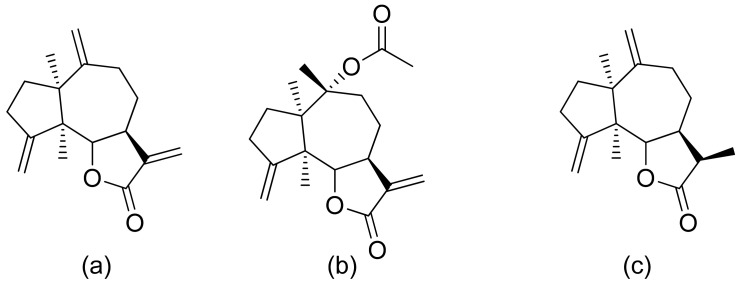
Sesquiterpene lactones from Portuguese liverwort (**a**–**c**).

Neves and colleagues tested the sesquiterpenoid lactones dehydrocostus lactone, acetyltriflocusolide lactone, and 11-αH-dihydrodehydrocostus lactone from Portuguese liverwort (Figure 3). All presented activity against *Cladosporium cucumerinum*, with the best result obtained for dehydrocostus lactone. This lactone was also the only lactone that presented activity against *C. albicans* [18].

**Figure 4:**
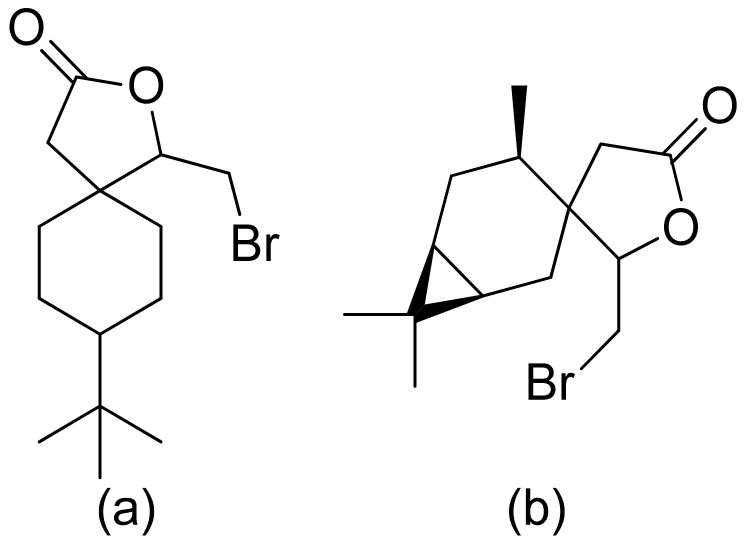
Bromolactone derivatives of monoterpenoids (**a**,**b**).

Kozioł et al. synthesized a 4-*tert*-butylcyclohexanone bromolactone derivative (Figure 4a), which was proven to inhibit the growth of *E. coli*, *S. aureus*, and *B. subtilis*. Additionally, at a concentration of 200 μg/mL, up to 60% of bacterial growth was inhibited [19]. Another previous study analyzed the antibacterial activity of bromolactone with a preserved carane system (Figure 4b). This bromolactone proved to have satisfactory growth inhibitory activity with an MIC90 value of 200 μg/mL [20].

**Figure 5:**
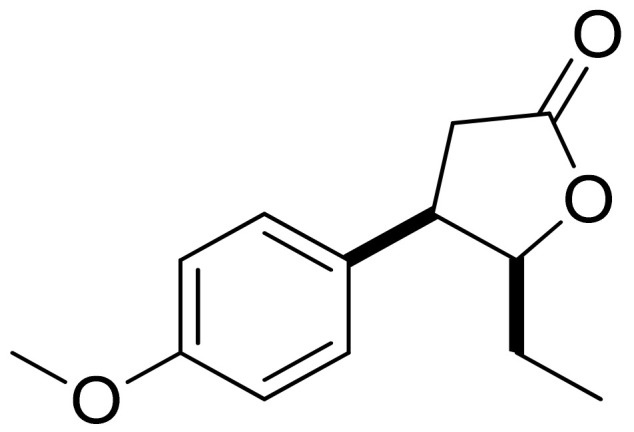
An anisaldehyde lactone derivative.

Mazur and colleagues synthesized anisaldehyde lactone derivatives and evaluated their antibacterial activity. One of the tested compounds (Figure 5) presented significant activity against two strains tested: *S. aureus* and *Listeria monocytogenes*. The MIC80 values were 50 and 100 ;µg/mL, respectively [21].

**Figure 6:**
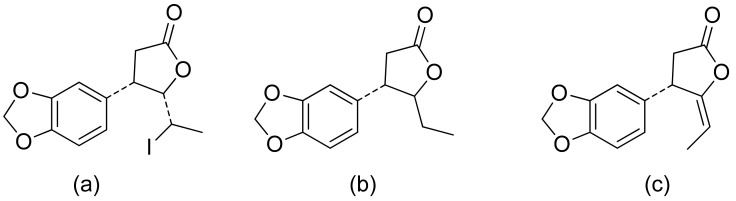
γ-Butyrolactones with a benzodioxol ring at the β-position (**a**–**c**).

A series of lactones were prepared from natural aromatic aldehydes by the Skrobiszewski group. All γ-butyrolactones were tested for their antifungal activity against four *Fusarium* strains, and three γ-butyrolactones (Figure 6) presented strong activity. The first derivative (Figure 6a) presented considerably high activity (50%) against *F. avenaceum* and *F. oxysporum*. The second lactone (Figure 6b) was the most active; against *F. oxysporum*, it showed 70% growth inhibition, and against *F. avenaceum*, *F. solani*, and *F. culmorum*, this lactone displayed 66%, 66%, and 55% growth inhibition, respectively. The third compound (Figure 6c) also exhibited approximately 50% activity against *F. avenaceum*, *F. solani*, and *F. culmorum*. Moreover, it was proposed that the reason for such results was that the most active compounds possess a benzodioxol ring at the β-position [22].

**Figure 7:**
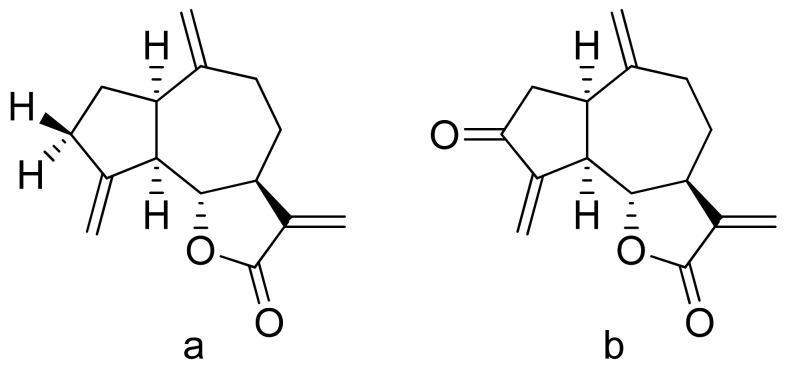
Lactones from the guajanolide class (**a**,**b**).

Terpenoid constituents from *Aglaia forbesii* seeds were obtained by the Joycharat group. Isoeichlerialactone, which is a derivative of 3,4-secodammarane, and a second lactone, a derivative of dammaran-3-one (isocabralealactone), were tested against *Phytophthora botryosa*, *Phytophthora palmivora*, and *Rigidoporus microporus*. Isoeichlerialactone was active against all phytopathogens tested and showing the best activity against *R. microporous* with a MIC and MFC (minimum fungicidal concentration) value of 62.5 µg/mL. However, isocabralealactone was active only against *R. microporous* with MIC and MFC values of 125 and 250 µg/mL, respectively [23]. Fernaández et al. verified the antifungal activity of lactones derived from *Hyalis argentea* var. latisquama against *Cryptococcus neoformans* and *C. albicans*. Lactone derivatives of lindenanolides were the most active. The lowest concentrations that obtained 100% inhibition were 62.5 μg/mL and 125 μg/mL, respectively, for one of the lactone derivatives [24]. The antifungal activities of natural and synthetic sesquiterpenoid lactones were tested against phytopathogens by Wedge and colleagues. Two compounds (Figure 7) had significant and moderate activities. One of the compounds (Figure 7b) at a 30 μM concentration reduced *Colletotrichum fragariae* growth by 90%, *Colletotrichum gloeosporioides* by 89%, and *Colletotrichum acutatum* by 29% but was not active against *Botrytis cinerea* or *F. oxysporum*. The other compound (Figure 7a) was the only compound active against *B. cinerea* with 22% inhibition at a concentration of 30 μM. It might be concluded that those compounds play a plant defense role in maintaining leaf integrity by inhibiting foliar pathogens [25].

Forville de Andrade and colleagues verified the antibacterial activity of a sesquiterpenoid lactone mixture extracted from yacon. It is a mixture of two lactones: enhydrin and uvedalin. The only microorganism susceptible to this mixture was *S. aureus* (ATCC 29213), and the MIC value was 750 μg/mL. The authors state that its activity might be related to its lipophilicity and weak polarity [26]. Costunolide and eremanthin isolated from *Costus speciosus* were not active against bacteria, although they proved satisfactory antifungal activity against *Trichophyton mentagrophytes, Trichophyton simii, Trichophyton rubrum 296, Trichophyton rubrum 57, Aspergillus niger, Epidermophyton floccosum, Curvularia lunata, Magnaporthe grisea*, and the MIC value were ranging from 31.25 to 250 μg/mL [27]. There is a relation between lactone structure and its antimicrobial activity, but further studies on the mode of action should have been undertaken. In many cases, its lipophilicity and low polarity support the antimicrobial activity of terpenoid lactones. It might be considered that as most plant secondary metabolites their main target is the cytoplasmic membrane and they can affect its structure and integrity, permeability, or functionality [28].

## 3. Cytotoxicity and Anticancer Activity

**Figure 8:**
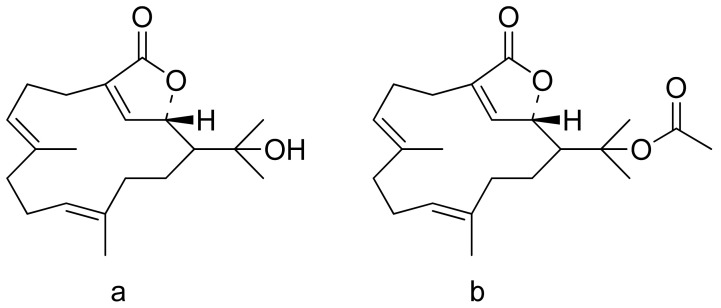
Terpenoid lactones from Formosan Soft Coral (**a**,**b**).

The therapeutic use of cytotoxic compounds or the drugs containing them might cause side effects. Thus, it is important to verify their impact on healthy cells. In contrast, cytotoxicity to cancerous cells might be able to be used as a therapeutic agent. Many lactones are known for their cytotoxicity in both healthy and tumor cells. Lactones occurring in nature become scaffolds for further synthesis of active compounds [29]. The cytotoxicity of artemisinin, a lactone of natural origin, was proven by Zheng. In this study, artemisinin showed activity against P-388 (mouse lymphocytic leukemia), A-549 (human lung carcinoma), and HT-29 (human colon adenocarcinoma) tumor cells with ED_50_ (the concentration that caused a 50% inhibition of cell growth) values ranging from 9.62 × 10^−2^ μg/mL to 4.41 μg/mL [30]. Choi et al. presented results on the cytotoxicity of terpenoid lactones from roots of *Ainsliaea acefifolia*. Among all of the extracted compounds they identified, were mokko lactone, zaluzanin C, and glucozaluzanin C. They tested their compounds in vitro against the following tumor cells: A549, SK-OV-3 (ovarian), SK-MEL-2 (skin melanoma), XF498 (CNS), and HCT15 (colon). The best result was obtained from zaluzanin C (ED_50_ = 0.36 μg/mL) and then by glucozalazuanin (ED_50_ = 0.40 μg/mL) in both skin melanoma cells lines. The remaining results ranged from 1.05 μg/mL to 2.73 μg/mL [31]. The Duh group tested the cytotoxicity of terpenoids from Formosan Soft Coral against A549, HT-29, KB (human epidermoid carcinoma), and P-388 cell cultures. Both lactones tested (Figure 8) were cytotoxic, but the cytotoxicity of the lactone acetate was significant: 3.03 μg/mL, 0.81 μg/mL, 0.72 μg/mL, and 1.20 μg/mL against the cell lines, respectively [32].

**Figure 9:**
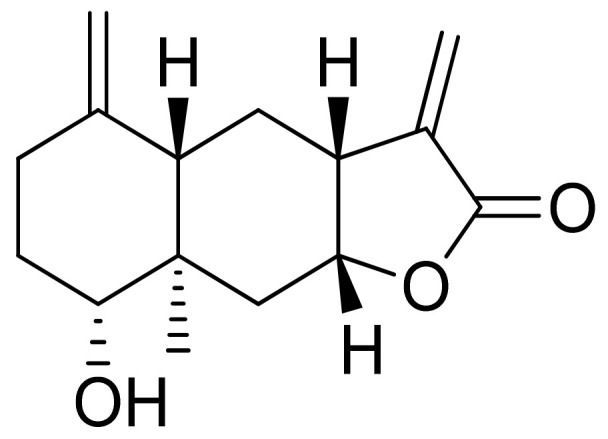
Asperilin.

Woerdenbag and colleagues analyzed the cytotoxicities of sesquiterpenoid lactones from *Arnica montana* flowers on the human carcinoma cells GLC-4 (lung small cell carcinoma) and COLO 320 (colon adenocarcinoma). Helenalin was far more cytotoxic than its esters. The lack of the ester group is probably responsible for its cytotoxicity, as there is an exocyclic methylene group fused to a lactone ring. Derivatives that did not have this group were 50–150 times less potent. Its cytotoxicity is correlated with the ability to undergo Michael-type reaction with biological nucleophiles [33]. Stojakowska et al. verified the cytotoxicity of the major terpenoids from *Telekia speciosa*. Asperilin (Figure 9) was moderately cytotoxic against PC3 (prostate carcinoma) cells in vitro (IC_50_ of 58.5 μM) and against melanoma cells (A375, WM793, and Hs294T) with IC_50_ values (the concentration that caused the death of 50% of cells) of 17.6 μM, 28.2 μΜ, and 29.5 μΜ, respectively. Authors recommend further studies on molecular mechanisms of action [34].

**Figure 10:**
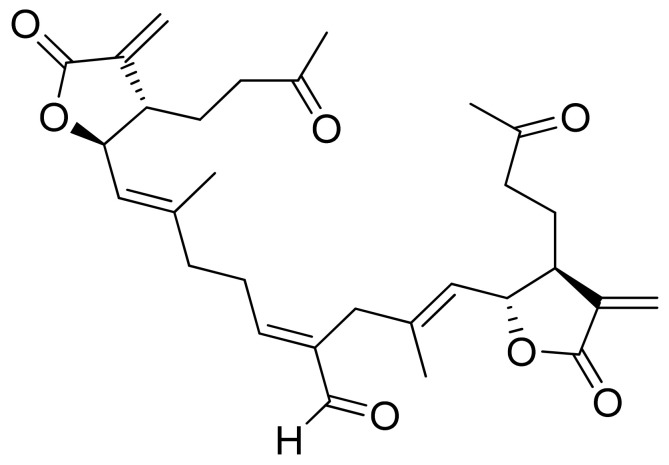
Kaunial.

Vernolides A and B, sesquiterpenoid lactones, were tested by the Kuo group against the KB, DLD1 (colon adenocarcinoma), NCI661 (lung large cell carcinoma), and HELA (cervical epithelioid carcinoma) tumor cell lines. Vernolide A was more cytotoxic (ED_50_ of 0.02, 0.05, 0.53, 0.04 μg/mL, respectively) than Vernolide B, which showed up to 180 times weaker cytotoxicity, further studies on structure-activity relation are recommended [35]. Maldonado and colleagues found a new terpenoid lactone from *Kaunia lasiophthalma* (Griseb.) and named it kaunial (Figure 10). Kaunial was significantly cytotoxic against the breast cancer lines L56BrC1 (IC_50_ = 0.98 μΜ) and SKBR-3 (IC_50_ = 1.6 μΜ), but also to healthy human cells. The presence of Michael acceptor is recognized as a factor that enhances its activity [36]. 

**Figure 11:**
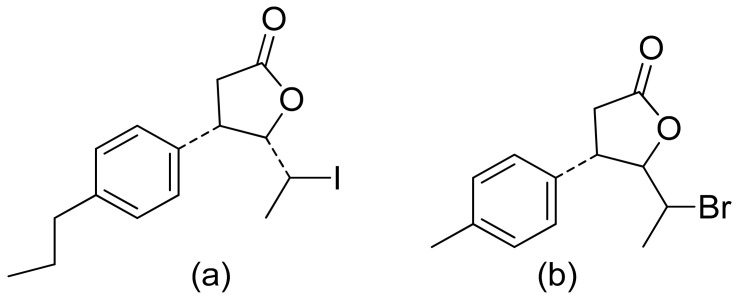
Iodo- (**a**) and bromolactone (**b**) from simple aromatic aldehydes.

Two C-11 terpene lactones (loliolide and isololiolide) were isolated from *Heliotropium bacciferum* Forssk. Their cytotoxicity was verified in the HCT116 (human colon cancer) and DLD1 cell lines. Both lactones showed IC_50_ values ranging from 0.306 to 0.351 mM in HCT116 cells and between 0.236 and 0.395 mM in DLD1 cells [37]. A wider group of lactones from the genus Sinularia proved to have cytotoxic activity rather than antimicrobial activity [16]. Both flexibilide and sinulariolide (Figure 2) are cytotoxic [38]. In vitro, sinulariolide was tested against the human KB, A-549, HT29, and P388 cell lines. The results were 7.6 μg/mL, 3.0 μg/mL, 3.1 μg/mL, and 3.9 μg/mL, respectively [39]. The same lactones as those from the antimicrobial test were evaluated by the Villarreal group against the KB, P388, and KBVI (vinblastine-resistant KB) cell lines. Lactones from the germacrolide and heliangolide groups were active against all of the cells, whereas eudesmane was moderately active against P388 cells [15]. Pawlak and colleagues synthesized two *trans*-β-aryl-δ-iodo-γ-lactone isomers from 2,5-dimethylbenzaldehyde and tested their cytotoxicity against CLB70 (chronic lymphoid leukemia), GL1 (acute canine lymphoid leukemia), and Jurkat (acute human lymphoid leukemia) cells. Both isomers showed 80% dead cells after treatment with 50 μg/mL solution, although the dextrorotatory isomer was more potent. Apoptosis was induced by a classic caspase-dependent pathway. Inhibition of two anti-apoptotic proteins (Bcl-xL and Bcl-2) was observed. Canonical apoptotic cell death was connected with phosphatidylserine exposure and caspase 3/7 activation. A decrease of the presence of anti-apoptotic proteins is followed by the activation of caspases and cleavage of PARP in the nucleus [40]. The antiproliferative activities of synthetic lactones towards similar cells, D17 (canine osteosarcoma cells) and CLBL1 (a canine B-cell lymphoma cell line) were tested by Gładkowski et al. Both stereoisomers of several compounds were evaluated, and the results showed that the *trans*-isomers were more active. The best results were obtained for (−)-*trans*-(4*S*,5*R*,6*S*)-5-(1-iodoethyl)-4-(benzo[d][1′,3′]-dioxol-5′-yl)dihydrofuran-2-one with IC_50_ values of 5.29 ± 0.31 μg/mL (Jurkat), 16.65 ± 2.56 μg/mL (D17), 5.08 ± 0.41 μg/mL (GL1) and 9.10 ± 0.96 μg/mL (CLBL1). Additionally, significant results were obtained for two other compounds, (−)-*trans*-(4S,5R,6S)-5-(1-iodoethyl)-4-(2′,5′-dimethylphenyl)dihydrofuran-2-one and its dextrorotatory form, with IC_50_ values ranging from 4.76 ± 0.52 μg/mL to 16.99 ± 4.88 μg/mL [41]. In his earlier study, other cytotoxic lactones emerged (Figure 11). Iodolactone (Figure 11a) displayed 83.7 ± 5.7% (Jurkat) and 35.2 ± 9.6% (D17) dead cells. Bromolactone (Figure 11b) resulted in 51.1 ± 8.9% (D17) and 47.6 ± 6.4% (Jurkat) dead cells [42].

The Lage group evaluated the antitumor activity of lactones against multidrug-resistant cell cancer lines: EPG85-257P (parental, drug-sensitive gastric carcinoma), EPG85-257RDB (gastric carcinoma with the classical MDR phenotype), EPG85-257RNOV (gastric carcinoma with an atypical MDR phenotype), EPP85-181P (parental, drug-sensitive pancreatic carcinoma), EPP85-181RDB (pancreatic carcinoma with the classical MDR phenotype), EPP85-181RNOV (pancreatic carcinoma with an atypical MDR phenotype), HT29P (parental, drug-sensitive colon carcinoma), HT-29RDB (colon carcinoma with the classical MDR phenotype) and HT29RNOV (colon carcinoma with an atypical MDR phenotype). The diterpenic α,β-unsaturated lactones helioscopinolide B, its acetylated derivative, and helioscopinolide E were effective, showing IC_50_ values of 5.7, 4.6, and 4.4 μM against the EPG85-257RDB cell line. The mechanism of action is related to the individual drug-resistant phenotype. The anticancer effects are not associated with a single factor, as a multimodal-mediated biological mechanism [43]. The nagilactone E a terpenoid isolated from *Podocarpus nagi* possesses anticancer activity towards lung cancer cells (A549). The mode of action of this group of compounds is omnidirectional. It is known that nangilactone E is a protein synthesis inhibitor [44]. It was also observed that it increases expression of PD-L1 (Programmed death-ligand 1) through activation of c-Jun (the protein encoded by JUN gene, the component of activator protein-1 pathway) and further leads to exposure to the plasma membrane of cancer cells [45]. Nimbolide is another lactone with potential in the treatment of cancer. It inhibits cell proliferation of MDA-MB-231 and MCF-7 (breast cancer) by inducing apoptosis signaling especially through activation of caspases and reduction proteins Bcl-2 which resulted in inhibiting cell progression and survival [46]. Against other breast cancer cells (TNBC) it also proved its activity by inhibiting cell growth via induction of apoptosis and anti-metastatic effects [47]. The same compound proved also activity to HONE-a cells (nasopharyngeal carcinoma) by inhibiting cell viability t induction of cell apoptosis via modulating extracellular signal-regulated kinases 1 and 2 and activation of caspases [48]. 

## 4. Anti-Inflammatory Activity

**Figure 12:**
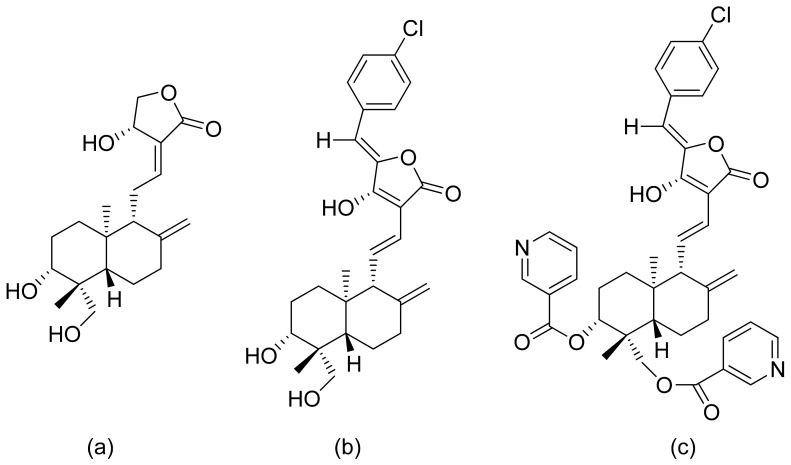
Andrographolide (**a**) and its derivatives (**b**,**c**).

Inflammation is a very complex phenomenon, and therapeutic agents might impact different aspects of this process. Inflammation is initiated by the secretion of proinflammatory cytokines such as interleukin 6 (IL-6), interleukin 8 (IL-8), and tumor necrosis factor (TNF-α), as well as the production of reactive oxygen species. Dai and colleagues revised the anti-inflammatory activity of andrographolide and its derivatives. They verified their ability to inhibit NO (nitric oxide) and PGE_2_ (prostaglandin E2) production, and also verified their impact on dimethylbenzene-induced mouse ear edema and egg albumin-induced rat paw edema. Three compounds (Figure 12) inhibited ear edema in mice at a dose of 0.90 mmol/kg body weight. Additionally, satisfactory results were observed against rat edema, with lactone c in Figure 12 showing the best reduction results at a dose of 0.90 mmol/kg body weight. All three compounds reduced PGE_2_ production at 1.35 mmol/kg and increased vascular permeability. At this same concentration, two compounds (Figure 12a,b) reduced NO production. Once again, compound c in Figure 12 obtained the best result at the dose of 0.90 mmol/kg. The great anti-inflammatory activity of compound c in Figure 12 is caused by the inhibition of iNOS activity and the reduction of NO production, moreover, it was the most potent α-glucosidase inhibitor [49].

**Figure 13:**
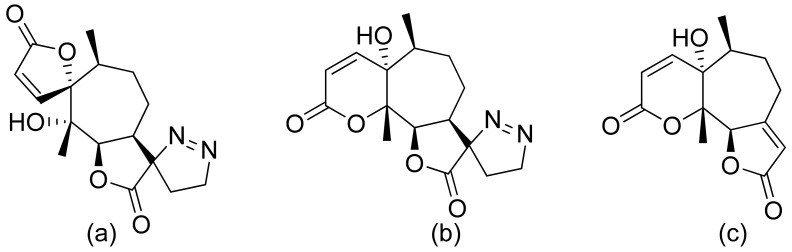
Psilostachyin derivatives (**a**–**c**).

In another study, the ability of andrographolide to inhibit LPS-induced (lipopolysaccharide-induced) TNF-α and IL-6 expression was tested. Inhibition of 62.54% lipopolysaccharide-induced TNF-α and 56% inhibition of IL-6 were observed. Additionally, other derivatives of this compound were synthesized and evaluated for their anti-inflammatory potential, proving that compounds with the 12-hydroxyl-14-dehydroandrographolide structure have higher inhibitory potential than those with isoandrographolide structures. The underlying mechanisms were not presented [50]. The Chib group synthesized psilostachyin, which is an acetylated pseudoguaianolide, and its derivatives and analyzed their anti-inflammatory potential by in vitro expression of TNF-α, IL-1β, and IL-6 in murine neutrophils. Three analogs (Figure 13) displayed good inhibitory effects on TNF-α cytokine secretion (a = 49.21%, b = 59.76% and c = 53.12%). Slightly worse results were obtained for the expression of IL-1β (a = 36.04%, b = 41.37% and c = 40.86%), and no significant results were determined for IL-6 (a = 11.32%, b = 14.01% and c = 17.06%). Both of these last two sets of results are expressed as the % inhibition against LPS [51].

**Figure 14:**
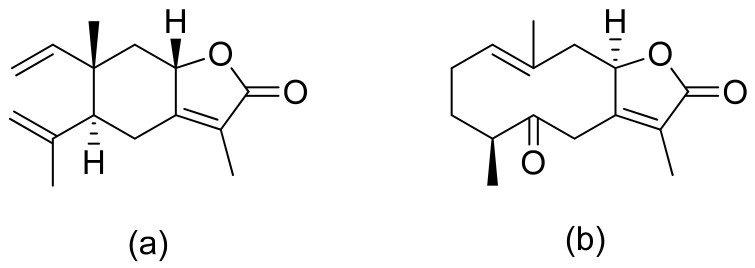
Sesquiterpenoid lactones from *Curcuma wenyujin* (**a**,**b**).

11α-13-Dihydrohelenalin ester derivatives from Arnica flowers were tested for their croton oil-induced mouse ear edema inhibitory activity. The percent inhibition ranged from 54% to 77% the underlying mechanisms were analyzed for Arnica flower tinctures and not for particular compounds. It was observed that stimulation of TNF-α induced DNA binding activity in Jurkat T cells–NF-κB p50/p65 heterodimer. Additionally, NF-κB DNA binding in an EMSA (electrophoretic mobility shift assay) and NF-AT (Nuclear factor of activated T-cells) DNA binding were weakened [52]. NF-κB (nuclear factor kappa-light-chain-enhancer of activated B cells) inhibitors were synthesized by Schorr and colleagues. It was proven that uvedalin inhibited NF-κB DNA binding at the concentrations of 2.5 μM in Jurkat T cells and 5.0 μM in RAW 264.7 cells (Abelson murine leukemia virus-induced tumor cells); moreover, no cytotoxic effects were observed. At a concentration of 10 μM, the NF-κB DNA binding process was inhibited by enhydrin. Both compounds inhibited the factor NF-κB transcription [53]. The impact of highly oxidized sesquiterpenoids from *Artemisia* was tested by Chi et al. Six terpenoids possessing an α-methylene-γ-lactone moiety proved their anti-inflammatory potential in a model of LPS-induced NO production in RAW 264.7 cells with IC_50_ values of 2.38–10.67 μM [54]. This same model was applied by Xia and colleagues for the analysis of sesquiterpenoids from the essential oil of *Curcuma wenyujin*. Two lactones, isogermafurenolid and curdionolide B (Figure 14), exhibited satisfactory inhibitory effects with IC_50_ values of 30.62 and 14.50 μM, respectively [55].

**Figure 15:**
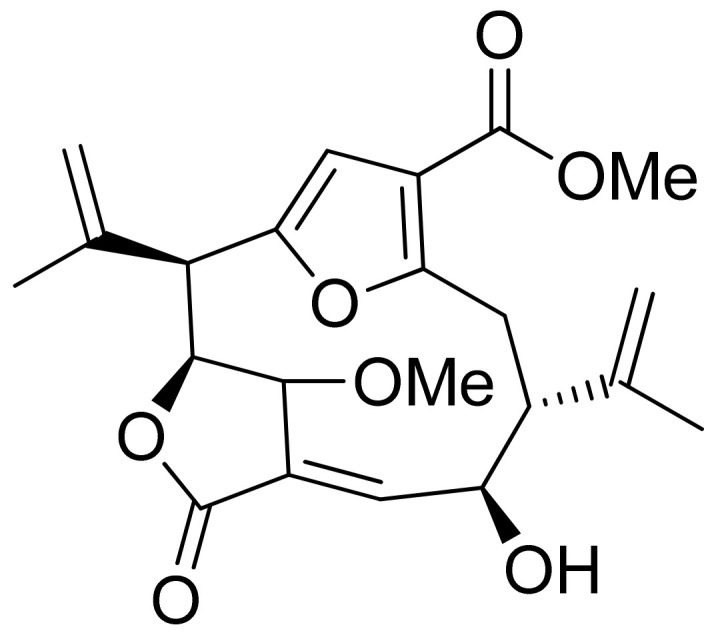
Pseudopterane diterpene**.**

A pseudopterane diterpene (Figure 15) was thoroughly examined for its anti-inflammatory potential. This compound not only decreased the production of the mediators TNF-α, IL-6, IL-1β, IP-10 (interferon γ-induced protein), iNOS (inducible oxide synthase), COX2 (cyclooxygenase 2), and MCP-1 (monocyte chemoattractant protein 1) induced by LPS in macrophages but also inhibited the degradation of IκBα (nuclear factor of kappa light polypeptide gene enhancer in B-cell inhibitor alpha) and the activation of NFκB, reducing the expression of the costimulatory molecules CD80 and CD86 in the LPS-induced process. All regulations might concern the transcriptional level [56].

Plaunolide is one of the components of the leaf crude extract of *Croton stellatopilosus*. Plaunolide was also proven to have significant inhibitory activity towards NO production in LPS-induced RAW 264.7 cells with an IC_50_ of 17.09 μM; moreover, it was nontoxic to cells. It can downregulate the expressions of the COX-1, COX-2, and iNOS genes [57]. Randainin D, a new diterpenoid found in *Callicarpa randaiensis*, exhibited mild inhibition of elastase release and moderate superoxide anion generation inhibitory activity of 35.9% at a concentration of 28.6 μM and an IC_50_ value of 21.5 μM [58]. New inositol lactones with anti-inflammatory potential isolated from *Inula montana* showed an impact on the release of NO on RAW 264.7 macrophages with IC_50_ values below 30 μM [59]. Shizukaol B is a lactone found in *Chloranthus henryi*, and this compound showed inhibition of inflammatory mediators such as iNOS and COX-2 after LPS stimulation. Anti-inflammatory effect is related to inhibition of expression of iNos and COX-2, blocking JNK (c-Jun N-terminal kinase) phosphorylation and c-Jun phosphorylation, attenuated c-Jun nuclear translocation. Moreover, shizukaol B inhibited the binding activity of AP-1 to DNA oligonucleotide [60]. From another plant from the Chloranthus genus, new chololactones were extracted. All of the compounds had a moderate activity with IC_50_ values of 4.4–35.4 μM [61]. Another study identified zaluzanin C as a potential inhibitory agent of NO production in RAW 264.7 macrophages with an IC_50_ value of 6.54 μM [62]. Nagilactones in a form of glucoside inhibit NF-κB activity what suppress LPS-induced NO production on RAW264.7 macrophages The phosphorylation of IKKα/β, IκBα, and p65 was reversed, by what the translocation of NF-κB/p65 from the cytoplasm to nucleus was prevented. Which resulted in the suppression of iNOS expression [63]. In addition, lactones ((4*S*)-hydroxy-(8)-methoxyl-(5*S*)-(H)-guaia1(10),7(11)-dien-12,8-olide and curcuminol G isolated from *Curcuma kwangsiensis* proved significant anti-inflammatory activity. They inhibited carrageenan-induced paw edema in vitro; at a concentration of 20 μg/mL they proved better inhibitory ratios on IL-1β and COX-2 than dexamethasone [64]. Michael and colleagues proved the inhibitory potential of rudbeckolide 5-LOX (lipoxygenase) by 84.9% at 10 μg/mL which might indicate that it would have the potential in preventing inflammation [65].

## 5. Antimalarial Activity

**Figure 16:**
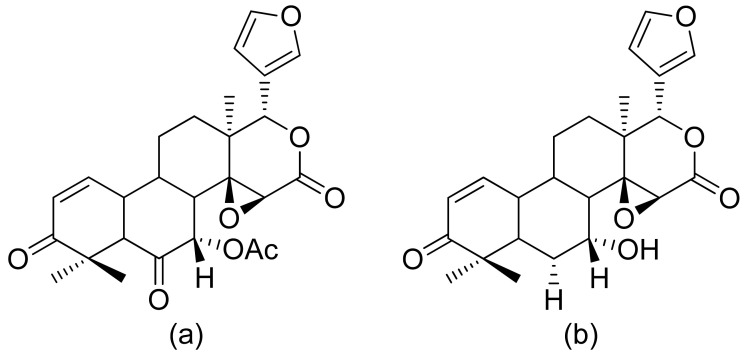
Antiplasmodial limonoids (**a**,**b**)**.**

Malaria is a disease that threatens human wellbeing and affects millions of people worldwide. This disease is caused by different species of the genus *Plasmodium* [66]. Artemisinin is the second treatment after quinine, and artemisinin derivatives are known as antimalarial compounds. This compound has been used as a drug since 1979, mostly in patients with chloroquine-sensitive or chloroquine-resistant strains of *P. falciparum* [67]. Its key pharmacophore is 1,2,4-trioxolane cycle spiro-conjugated with sesquiterpene δ-lactone [68]. The Pereira group tested limonoids and their derivatives from biomass after the production of andiroba oil for their in vitro and in vivo antiplasmodial activity. 6α-Acetoxygedunin and 6α-hydroxydeacetylgedunin (Figure 16) possessed the best in vitro activity against the *P. falciparum* K1 strain, with IC_50_ values of 7.0 and 5.0 μM, respectively. Compound a in Figure 16 was also tested in vivo in a rodent malaria model against *P. berghei* NK65. Compared to untreated animals, 65.7% suppression of parasitemia was observed at the oral dose of 100 mg/kg/day [69].

**Figure 17:**
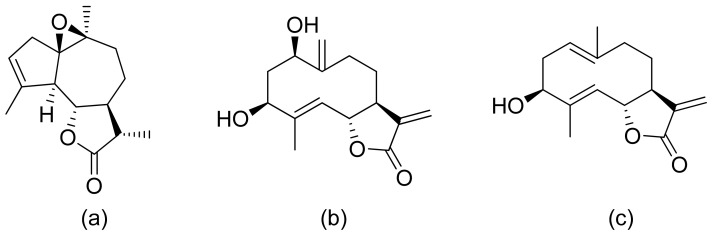
Antiplasmodial compounds from *Artemisia gorgonum* (**a**–**c**).

The Moon team analyzed the antimalarial activity of ineupatorolide A isolated from *Carpesium rosulatum*. In his first study, he proved its activity in vitro against a chloroquine-sensitive strain of *P. falciparum* (D10), resulting in an IC_50_ value of 0.007 μg/mL [70]. Then, the same compound was tested in vivo against *P. berghei* in mice. In the first 4 days of infection, the dosages of 2, 5, and 10 mg/kg/day showed blood schizontocidal activity (63.2–86.5% suppression). The authors suspect that there are two mechanisms behind the antimalarial activity–elevation of erythrocytic oxidation or/and inhibiting protein synthesis [71]. (+)-4′-Decanoyl-*cis*-khellactone and (+)-3′-decanoyl-*cis*-khellactone were isolated from *Agelica purpuraefolia*. Both compounds showed notable antiplasmodial activity against the D10 strain with IC_50_ values of 1.5 and 2.4 μM, respectively [72]. Tagitinin C was tested for its antiplasmodial activity in vivo against *P. falciparum* with an IC_50_ value of 0.33 μg/mL [73]. Another in vitro study was performed by Kraft and colleagues, and five lactones proved to be active against the chloroquine-sensitive strain of *P. falciparum* PoW and the chloroquine-resistant clone Dd2. 1-Desoxy-1a-peroxy-rupicolin A-8-*O*-acetate, rupicolin A-8-*O*-acetate and 1a,4a-dihydroxybishopsolicepolide showed moderate activity (IC_50_ values = 8.7, 12.5, 8.6 μg/mL against PoW; 17.5, 10.8, 11.7 μg/mL against Dd2, respectively). Vernodalol and its derivative 11b,13-dihydrovernodalin were more active (IC_50_ = 4.0 and 2.3 μg/mL against PoW; 4.8 and 1.1 μg/mL against Dd2, respectively) [74]. Pseudoguaianolide sesquiterpenoid lactones (helenalin and its derivatives) proved to be active against the asexual erythrocytic stages of *P. falciparum*, in vitro, with IC_50_ values of 0.23–7.41 µM; the best result was obtained with helenalin [75]. Oret et al. presented studies on sesquiterpenoid lactones, among which arborescin, ridentin, and hanphyllin (Figure 17) were significantly active against *P. falciparum* FcB1 with IC_50_ values ranging from 2.3 to 5.4 μg/mL [76].

**Figure 18:**
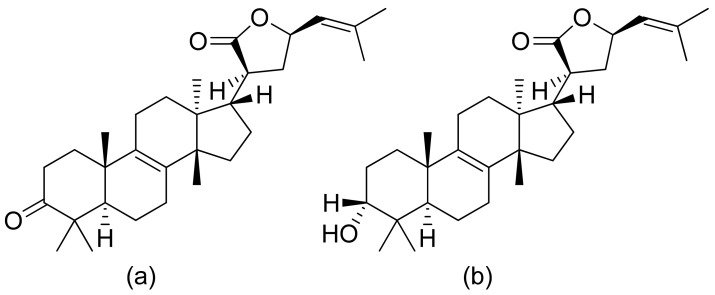
Triterpenoid lactones from the oleo-gum-resin of *Boswellia serrata* (**a**,**b**)**.**

Pedersen and colleagues presented another in vitro active lactone against *P. falciparum* strain D10 and the chloroquine-resistant strain W2. The IC50 values ranged from 1.55 to 3.82 μM against the D10 strain and 2.10 to 4.94 μM against the W2 strain [77]. Next, a sesquiterpenoid lactone (urospermal A-15-*O*-acetate) was isolated and analyzed for its antiplasmodial activity against *P. falciparum* strains 3D7 and W2. In vitro studies were performed, resulting in IC50 values of 2.87 and 2.41 μM, respectively [78]. The Sawadjoon group presented a spirodihydrobenzofuran terpenic lactone that had excellent in vitro activity against *P. falciparum* (K1, multidrug-resistant strain), obtaining an IC50 value of 0.15 μg/mL [79]. Tetranortriterpenoids, domesticulides B–D, and five triterpenoids were tested against this same strain as in a previous study and exhibited antimalarial activity with IC50 values of 2.4–9.7 μg/mL [80]. Triterpenoid lactones (Figure 18) isolated by Greve et al. from oleo-gum-resin of *Boswellia serrata* presented IC_50_ values of 1.0 µg/mL and 1.9 µg/mL against the chloroquine-sensitive *P. falciparum* NF54 strain [81]. 

Twelve new cassane-type diterpenes were isolated by the Guoxu group. Two lactones proved to possess antimalarial activity against the K1 strain with IC_50_ values of 0.78 and 0.52 μM [82]. Graziose et al. presented the antiplasmodial activity results of compounds isolated from plants. Peroxyferolide and lipiferolide were tested against the D10 strain (IC_50_ values of 6.2 and 1.8 µg/mL, respectively) and Dd2 strain (IC_50_ values of 12.7 and 7.5 µg/mL, respectively). The authors suspect that the antimalarial activity of the peroxide is due to the hydroxy peroxide group, which is chemically related to the endoperoxide residue of artemisinin to which it owes its activity [83]. 2-methoxyisogermafurenolide and 8-*epi*-2-methoxyisogermafurenolide were isolated as a mixture from Myrrh. A combination of those two sesquiterpenoid lactones showed antiplasmodial activity against *P. falciparum* with an IC_50_ value of 2.9 mg/L [84]. 

## 6. Antifeedant Activity

**Figure 19:**
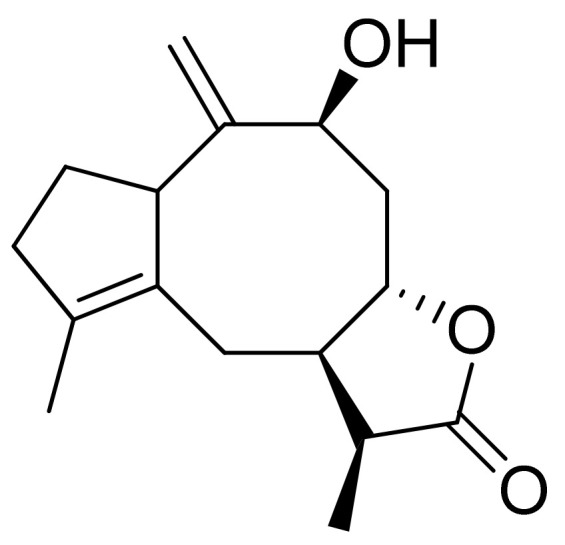
Antifeedant lactone from the fruits of *Carpesium abrotanoides**.***

Pests are a serious threat to crops, and it is important to control their feeding habits instead of controlling their population by extermination, which might disturb the ecological balance. Terpenoid lactones are well-known antifeedant agents. The genus *Helianthus* is rich in chemicals that serve as feeding deterrents for various pests. Argophyllin A, argophyllin B, and 4,5-dihydroniveusin A were isolated from sunflowers and were found to possess antifeedant activity against WCR (west corn rootworm), with relative rates of consumption at a concentration of 40 µg per disk after 48 h of 0.53, 0.75, and 0.90. The compounds interact with receptor sites on the gustatory sensilla of WCR [85]. Wu and colleagues tested sesquiterpenoid lactones on their twenty-four-hour antifeedant activity against 3rd instar larvae of *Plutella xylostella*. One lactone (Figure 19) presented excellent activity with an EC_50_ (the effective dosage for 50% feeding reduction) of 19.84 mg/L [86]. 

**Figure 20:**
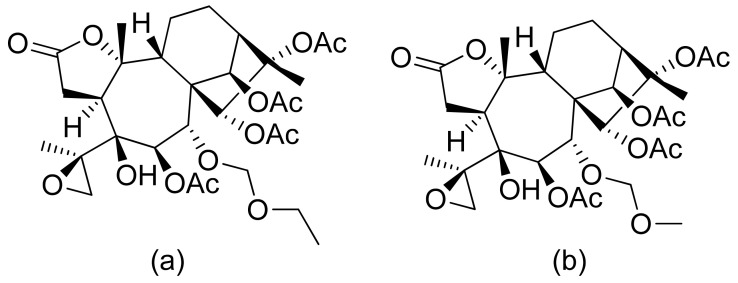
Antifeedant lactones from *Pieris Formosa* (**a**,**b**)**.**

A group of sesquiterpene lactones was extracted from *Vernonanthura nebularum*, four of which were obtained in good yields and tested for their antifeedant activity against *Spodoptera frugiperda* larvae. The results are presented as the antifeedant index, which is the ratio of consumption between treated and untreated disks. All tested lactones presented values above 50% [87]. Li et al. tested two diterpenoid lactones (Figure 20) isolated from the poisonous plant *Pieris formosa*. Both exhibited antifeedant activity against cotton bollworms (*Helicoverpa armigera*). The antifeedant index was 54.9% at a concentration of 5.54 × 10^−3^ μg/mL for compound a in Figure 20 and 40.7% at a concentration of 5.67 × 10^−3^ μg/mL for compound b in Figure 20 [88].

**Figure 21:**
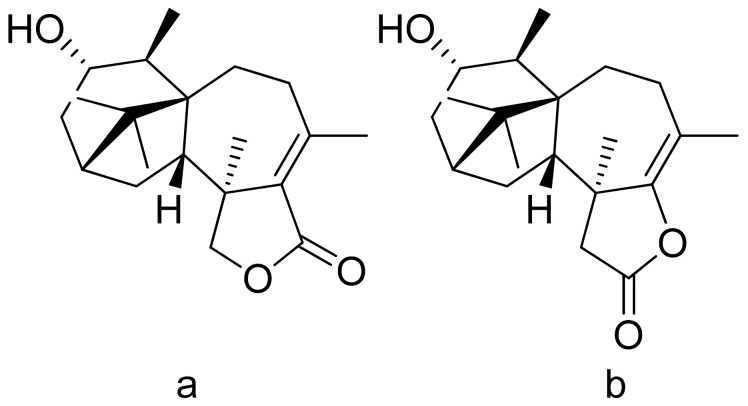
Harzianelactones A (**a**) and B (**b**).

Neopierisoids A and B, which are other grayanane diterpenoid lactones, were tested for their feeding inhibitory activity against *P. brassicae*, a plant-feeding generalist insect herbivore. These compounds presented EC_50_ values of 10.07 μg/mL and 5.33 μg/mL, respectively [89]. The feeding deterrent activity of synthetic lactones was widely studied by a group of Wawrzeńczyk. The anisaldehyde lactones mentioned before were studied for their feeding deterrent activity against *Sitophilus granaries* L., *Trogoderma granarium* Everts, and *Tribolium confusum* Du Val. The total coefficients of deterrence (T coefficients) are one of the parameters that characterize deterrence and are the sum of attractancy and repellence. The T coefficients value ranges between –200 and +200 when values are negative it indicates attractancy. T-coefficients were presented, and the results ranged from 143.3 to 183.9 [21]. In another study, the activity of a β-cyclocitral lactone derivative was verified against these same pests. The best result was obtained by chlorolactone, with a T coefficient of 160.8 [90]. γ-Butyrolactones that possess antifungal activity were also tested for their antifeedant activity against storage pests: the larvae and adults of confused flour beetles (*Tribolium confusum*), the larvae of khapra beetles (*Trogoderma granarium*), and the adults of granary weevils (*Sitophilus granarius*). The best result was obtained against khapra beetles by one γ-ethyl-γ-lactone, resulting in a T coefficient of 167.9 [22]. Racemic β-aryl-γ-ethylidene-γ-lactones proved to have feeding inhibitory activity against both the adults and larvae of *Alphitobius diaperinus* Panzer, resulting in T coefficients ranging from 72.60 to 185.52 [91]. Three (+)-3-carene derivatives (Table 1) were tested for their feeding deterrent activity on the storage pest insects mentioned before. One of the lactones (Figure 21) exhibited moderate activity with a T coefficient of 120.9 for *Trogoderma granarium* [92]. 

Additionally, bromolactone (Figure 4a) was tested against dangerous pests, the lesser mealworm *Alphitobius diaperinus* (Panzer), and the peach potato aphid *Myzus persicae* (Sulzer); unfortunately, its deterrent activity was low against aphids and adult mealworms, and it was even an attractant to mealworm larvae [20]. Feeding of *Schistocerca americana* is reduced by tomentosin and xanthinosin as its concentration increased. Although at the same concentration *trans* form of lactone reduced consumption. As sesquiterpenoid lactones interfere with various processes and molecular targets it is difficult to identify a specific one. Authors suspect that the molecular target is GABa/glycine sensitive receptors which leads to chemical disruptions of neurons [93].

## 7. Other Biological Activities

Terpenoid lactones possess various biological activities that are not extensively studied, although their considerably potential is worth mentioning. Phytotoxicity is observed for various lactones from the terpenoid group. Control of weeds is a very important aspect of agriculture and the use of compounds of natural origin is a great alternative for traditional methods. Inuloxins A, C, D isolated from *Inula viscosa* proved the ability to inhibit seed germination of crenate broomrape and field dodder at a concentration of 4.0·10^−4^ M and 1.6·10^−3^ M, respectively [94]. Harzianelactones proved phytotoxic activity towards seedling growth of amaranth and lettuce. Harzianelactone A (Figure 21a) inhibited root and hypocotyl growth of amaranth at a concentration of 200 ppm, and a concentration of 50 ppm significantly decreased growth of both plant parts. Towards lettuce hypocotyl better results were observed for herzianelactone B at concentration 200 ppm, it reduced hypocotyl elongation [95].

**Figure 22:**
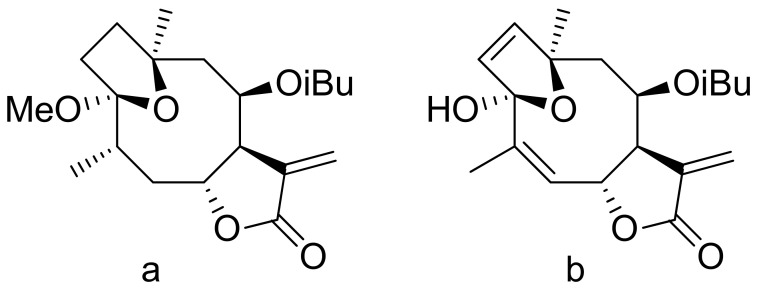
Antileishmanicidal lactones from *Tithonia diversifolia* (**a**,**b**)

Xu and colleagues demonstrated that diterpenoid momilactones are involved in allelopathy. They applied reverse genetics by knocking out relevant diterpene synthases to prove it. Rice due to the presence of momilactones can suppress the growth of other plants in its neighborhood [96]. In addition to antiplasmodial activity terpenoid lactones present activity towards other parasites. De Toledo et al. presented antileishmanicidal activity of sesquiterpenoid lactones from *Tithonia diversifolia*. Tagitinin F and one of the 1,10-epoxy heliangolides (Figure 22a,b) obtained good LD_50_ values against *Leishmania brasiliensis* (13.7 ± 2.6/37.4 ± 7.1 µg/mL/µM and 7.4 ± 2.8/21.2 ± 8.0 µg/mL/µM, respectively) and relevant selectivity index. Both were then extensively tested against intracellular parasites by peritoneal macrophages and were able to reduce the infection index [97].

Otoguro and his group proved antitrypanosomal activity against *Trypanosoma brucei* of sesquiterpenoid lactones. The best result was obtained by 4-peroxy-1,2,4,5-tetrahydro-α-santonin with the EC_50_ value 0.40 ± 0.02 µg/mL [98]. Enzyme inhibition is a very important target in drug design. Sester-terpenoid YW3548–lactone isolated from *Paecilomyces formosus* LHL10 proved inhibitory activity towards urease and α-glucosidase at concentration 250 µg/mL. Such activity might indicate possible future use as a drug for enzyme inhibition [99]. Andrographolide (Figure 12a) proved hepatoprotective activity in paracetamol-induced liver injury in mice. Ex vivo studies proved that andrographolide especially in form of nanocrystals preserves native hepatocellular structures [100]. This compound also indicates thrombolytic activity. Prakash et al. proved andrographolide lyses blood clots in vitro [101].

## 8. Conclusions

In conclusion, lactones have been indicated to possess various biological activities that are often directly connected with lactone moieties. Artemisinin is already being used as an antimalarial drug. Many newly synthesized lactones and those isolated from plants have shown promising properties, such as antimicrobial, anti-inflammatory, anticancer, and antiplasmodial activities, as well as potent feeding deterrents. Unfortunately, antioxidant activity is not in the domain of the activity of lactones. Consequently, further studies on lactones and their biological activities should be undertaken to determine additional active agents, and it might also be beneficial to consider these compounds as antioxidants to verify this inactivity. Additionally, in many cases, molecular targets of terpenoid lactones remain unknown. Both the field of chemical synthesis of new lactone derivatives and the expansion of research on already known lactones should be explored for further in vivo studies.

## Figures and Tables

**Table 1:** Feeding deterrent activity of lactones with carane system.

Storage Pests Insects	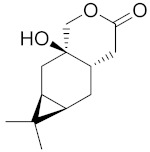	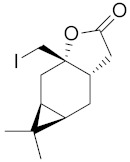	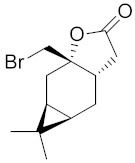
Confused flour beetle—larvae (*Tribolium confusum* Duv.)	55.0	37.8	108.1
Confused flour beetle—adults (*T. confusum* Duv.)	48.8	84.2	93.7
Granary weevil beetle—adults (*Sitophilus granarius* L.)	107.3	53.9	104.0
Kharpa beetle—larvae (*Trogoderma granarium* Ev.)	52.3	7.5	120.9

## Data Availability

Not applicable.

## Abbreviations

BVMOs	Baeyer–Villiger monooxygenases
MIC	Minimal inhibitory concentration
MFC	Minimal fungicidal concentration
ED_50_	The concentration that caused a 50% inhibition of cell growth
IC_50_	The concentration that caused the death of 50% of cells
LPS	Lipopolysacharide
IL-6	Interleukin 6
IL-8	Interlekin 8
TNF-α	Tumor necrosis factor
NO	Nitric oxide
PGE2	prostaglandin E2
NF-κB	Nuclear factor kappa-light-chain-enhancer of activated B cells
IP-10	Interferon γ-induced protein
iNOS	Inducible oxide synthase
COX2	Cyclooxygenase 2
MCP-1	Monocyte chemoattractant protein 1
IκBα	Nuclear factor of kappa light polypeptide gene enhancer in B-cell inhibitor alpha
WCR	West corn rootworm
EC_50_	The effective dosage for 50% feeding reduction
T coefficients	The total coefficients of deterrence
